# The Diagnosis of Appendicitis

**Published:** 1902-07-12

**Authors:** 


### THE DIAGNOSIS OF APPENDICITIS.

Among the many interesting matters referred to by Sir Frederick Treves in the Cavendish lecture were two which are of daily importance to the practitioner, namely, the significance of localised tenderness in appendicitis and the palpation of the diseased process itself. In the literature of appendicitis great importance, he says, is attached to tenderness over a certain limited area called McBurney's point. With many this is an inspired sign, a diagnostic talisman, a key to the whole clinical position. If there be tenderness at this spot then the affection is appendicitis; if much tenderness be not perceived then there is a strong presumption that no appendicitis is present. The symptom thus becomes the very touchstone of the disease. Moreover, it is assumed by many that this tenderness points to the site of the diseased process, or that it at least indicates the centre of the involved district. "I believe," writes Dr. McBurney, "that in every case of appendicitis the seat of greatest pain determined by the pressure of one finger, has been very exactly between $1\frac{1}{2}$ and 2 inches from the anterior spinous process of the ilium in a straight line drawn from that process to the umbilicus. The point corresponds very accurately in the living subject to the base of the appendix." He considers that tenderness at this spot gives evidence of the disease during the first few hours of the attack, and he adds, "no other acute disease presents this feature." It is needless to say, remarks Sir Frederick, that tenderness in the right iliac fossa is a very conspicuous symptom of appendicitis of all grades, and as the "point" under discussion is about the centre of this fossa it may be the centre of the tender area, but beyond this he does not think that the sign in question is of any clinical value. It does not serve to indicate either the starting point of the disease or even its chief point. It does not indicate the situation of the diseased appendix, nor does it even correspond in the subject with the base of the appendix, and so far from its being peculiar to appendicitis tenderness at this point is common in healthy persons, while in subjects of colitis involving the cæcum such tenderness may be quite acute. With the object of elucidating the anatomical questions involved Sir Frederick Treves asked Dr. Arthur Keith to investigate the matter, and he has been able to show (*a*) that the two iliac fossæ present no anatomical differences except that the cæcum is found on the one side and the sigmoid flexure on the other; (*b*) that the structure peculiar to the right side which comes more or less precisely under McBurney's point is the ileo-cæcal valve, while the base of the appendix lies on an average rather more than an inch below the opening of the ileum; and (*c*) that there seems to be but little doubt that the right-sided tender spot which can be made out in so large a proportion of healthy individuals is represented by the ileo-cæcal valve. The tenderness referred to is well-defined, is elicited by deep pressure with the finger, and is represented by no corresponding sensitiveness on the left side. In some cases of colitis, notably when the trouble is on the right side and chronic the tenderness at McBurney's point is very marked.

So much for the diagnostic, and indeed pathogonomic, value attached by so many to tenderness at this spot. Sir Frederick Treves then proceeds to discuss another source of error in the diagnosis of appendicitis. In palpating the abdomen above the iliac fossa in a patient suspected of appendicitis, he says that an elongated body can sometimes be felt which is often mistaken for a swollen appendix. The little tumour is pipe-like and is either vertical, or more usually is placed obliquely. Over and over again the discovery has been announced of a diseased appendix lying vertically or obliquely in the iliac fossa. This, however, is a phantom tumour, and it may generally be safely asserted that when the part is exposed by operation the diseased organ will not be found to occupy the site of the elongated body. Experience induces a great suspicion of that diseased appendix which is stated to be placed vertically or nearly so, and which is so readily felt. This phantom is undoubtedly due to muscular contraction, sometimes of the outer edge of the rectus muscle, sometimes of fibres of the internal oblique or transversalis muscles, and is probably connected with the fact that the bowel, the parietal muscles over it, and the skin over them again, are all supplied by branches of the same spinal nerve.

